# Influence of Perfluoroalkyl Substances on Occurrence of Coronavirus Disease 2019

**DOI:** 10.3390/ijerph19095375

**Published:** 2022-04-28

**Authors:** Zygmunt F. Dembek, Robert A. Lordo

**Affiliations:** Battelle Memorial Institute, Columbus, OH 43201, USA; lordo@battelle.org

**Keywords:** polyfluoroalkyl substances, PFAS, PFOA, PFOS, COVID-19

## Abstract

Epidemiologic evidence indicates exposure to polyfluoroalkyl substances (PFAS) influences immunosuppression, with diminished vaccination response. The relationship between PFAS blood levels and coronavirus disease 2019 (COVID-19) occurrence by age warrants further examination. This assessment identified blood PFAS exposure levels in discrete populations. Recent PFAS population studies summarizing age and gender results were identified and included. Geographically corresponding COVID-19 incidence data were determined for selected counties in North Carolina (NC) and Ohio (OH), and the state of New Jersey (NJ). Centers for Disease Control and Prevention COVID-19 databases were accessed for national incidence data by age groupings. We assessed associations between blood PFAS concentrations, COVID-19 incidence rates, and key demographic characteristics, within subpopulations. COVID-19 incidence counts and blood PFAS concentration were obtained for each age group, along with estimated U.S. Census total population. A general trend observed is higher PFAS levels in older age groups. Younger age groups contained fewer COVID-19 cases. Global COVID-19 mortality is highest in elderly populations with hospitalization and death greatly increasing from age 50. PFAS exposures occurring early in life may cause deleterious health effects later in life, including decreased antibody response and reduced disease resistance. Highest levels of both PFAS exposure and COVID-19 were found in the oldest populations. While this does not determine causality, such associations should help promote further study.

## 1. Purpose

The primary objective of this study is to review recent existing published data on concentrations of polyfluoroalkyl substances (PFAS) isomers in blood within specified segments of the U.S. population and to assess the extent of association with coronavirus disease 2019 (COVID-19) incidence rates (confirmed cases attributed to COVID-19).

## 2. Introduction

Elevated levels of PFAS isomers in human biological fluids such as blood have been linked to adverse effects on the human immune system, including a reduced immune response to vaccines [1]. As a result, U.S. government health agencies such as U.S. Centers for Disease Control and Prevention (CDC) and Agency for Toxic Substances and Disease Registry (ATSDR) wish to better characterize the putative relationships between PFAS levels in human biological fluids and organs, and the prevalence of COVID-19 cases in the U.S. population [2]. Preliminary studies indicate that history of significant PFAS exposure could place individuals at risk for severe COVID-19 illness, but more studies are needed [3]. A recent study of blood samples from a Danish cohort (*n* = 323) of COVID-19 patients from ages 30–70 years found that elevated levels of plasma PFAS (perfluorobutanoic acid (PFBA) in particular) were associated with an increased risk of achieving a severe case of COVID-19 [4]. These researchers hypothesized that elevated PFAS levels in blood result in immune suppression and a decreased response to vaccines.

This preliminary study examines existing data on blood PFAS levels and on COVID-19 incidences coexisting in the same municipal populations within the United States, to determine the extent of association between them and how this association may be age-related. It is preliminary in that it relies on blood PFAS summary measures within subpopulations in specific locations of the country as reported in existing publications, and the ability to match these measures with COVID-19 incidence rates for those same locations and subpopulations. The number of previous PFAS studies whose results could be linked to community COVID-19 infection data is currently limited. In addition, data from previous cohort studies or from Government human health databases that measure both blood PFAS concentrations and COVID-19 incidences in the same individuals are not yet publicly available.

## 3. Materials and Methods

### 3.1. Data Acquisition

#### 3.1.1. Blood PFAS Data

PubMed and Google Scholar databases were searched for publications that summarized blood PFAS concentrations measured in the National Health and Nutrition Examination Survey (NHANES) which were representative of the nation or for specific demographic groups. Early release tables providing summary statistics on PFAS isomers in human serum from the NHANES were identified and obtained from CDC’s website [5]. In addition, from these databases, we identified publications from the recent five years of research studies and health surveys in which blood PFAS measurement summaries were reported for study participants in demographic groups including age, gender, and race/ethnicity. While publications for studies in various countries were identified, we were interested only in studies involving individuals residing in communities within the United States. We identified PFAS studies that occurred in the following areas: Ann Arbor, Michigan; Boston, Massachusetts [6]; Cincinnati, Ohio [7]; Davis-Sacramento, California; the Fernald Superfund area in Butler County, Ohio [8], Minneapolis-St. Paul, Minnesota [9]; Oakland, California; Pittsburgh, Pennsylvania; San Francisco, California [10,11]; Wilmington, North Carolina [12,13], and Urbana-Champaign, Illinois [11], and a publication summarizing PFAS levels in residents across the state of New Jersey [14]. Each publication considered a different set of PFAS isomers and defined demographic groups of participants differently. While each publication reported on blood PFAS concentrations, three summarized these measurements in tables according to demographic factors, which were required for use in this investigation. These were for selected counties in North Carolina [12] and Ohio [7], and the state of New Jersey [14].

Only PFAS measurement summaries presented in tables within these publications were available for this analysis (i.e., measurements at the individual level were not available). These summaries included distributional statistics such as geometric mean and selected percentiles (e.g., 25th, 50th, 75th, 95th) calculated across the research study’s participants according to demographic group. Similar to the demographic group definitions and the set of PFAS isomers measured, the set of distributional statistics presented in the summary tables differed from one publication to another.

The focus of PFAS data collection from these studies was on the isomers perfluorooctanoic acid (PFOA, also known as C8) and perfluorooctanesulfonic acid (PFOS). Typically, PFOA and PFOS make up the largest percentage of total PFAS concentration and are often the two PFAS isomers assessed in public health studies. This is primarily because PFAS and PFOA were first commercially manufactured in the late 1940s and early 1950s, and were once found in many commercial products such as Teflon^TM^ and Scotchgard^TM^. Thus, when total PFAS concentration is in the detectable and reportable range for a given sample, these two isomers tend to be most likely detected and therefore reported.

#### 3.1.2. COVID-19 Incidence Data

For this analysis, publicly available COVID-19 incidence data (i.e., number of diagnosed cases and deaths, and/or percentages relative to the total population, both overall and within specified demographic groups) were obtained for those locations represented in the PFAS study publications which we identified (i.e., states or regions within states). These data were downloaded from COVID-19 data dashboards typically operated by State health departments.

Each COVID-19 data source defined a COVID-19 case in the same way: according to case definitions defined by the CDC [15]. They often differed, however, in the types and definitions of demographic groups used in the data reporting. Other differences include how often they updated their incidence data, whether data are available on a daily basis through the pandemic or only cumulative across this period, and whether they report data by county or only over the entire state within the demographic groups. As a result of differences such as these, and due to the different types of COVID-related restrictions in place at different locations and when they were implemented, any comparison of COVID-19 incidence data among different states and cities must be conducted with caution. We also accessed COVID-19 databases made available by the CDC on COVID-19 incidence data for the entire U.S. by age groupings.

#### 3.1.3. Population Count Data

For this investigation, COVID-19 incidences in a particular location were expressed as a percentage relative to the total estimated population for the given demographic group. To convert numbers of COVID-19 incidences to percentages, we obtained total population estimates for U.S. counties, overall and within gender and age groups, from the U.S. Census Bureau website [16]. The estimates represent the total population as of 1 July 2019, and correspond to estimates from the 2010 Census plus any adjustments made by the U.S. Census Bureau to update the estimates to 2019.

At the national level, we obtained U.S. population estimates for 2010 through 2019 by gender and age, where age is expressed as a specific year (rather than a range of years). Population data for 2019 were used to calculate the COVID-19 incidence percentages used in this investigation.

### 3.2. Methods

Across the identified studies that reported on concentrations of PFAS isomers in blood, this investigation focused on assessing the level of association between measures related to the distribution of PFAS concentrations in blood within the subpopulations targeted in those studies and COVID-19 incidence rates in those study locations. As data on both PFAS blood concentration and COVID-19 incidence are not yet publicly available for a sufficient-sized cohort of individuals in the United States, the investigation assessed this association at the location and subpopulation level, and not the individual level. This investigation also considered how this association may differ according to age within the subpopulations. Age was considered here as it was the most frequent demographic encountered between blood PFAS concentrations in the selected studies and COVID-19 incidence from the state databases. PFAS summary statistics and COVID-19 incidence data were less frequently reported for other potentially important population demographic groups such as race/ethnicity, socio-economic status, and gender.

**Figure 1:**
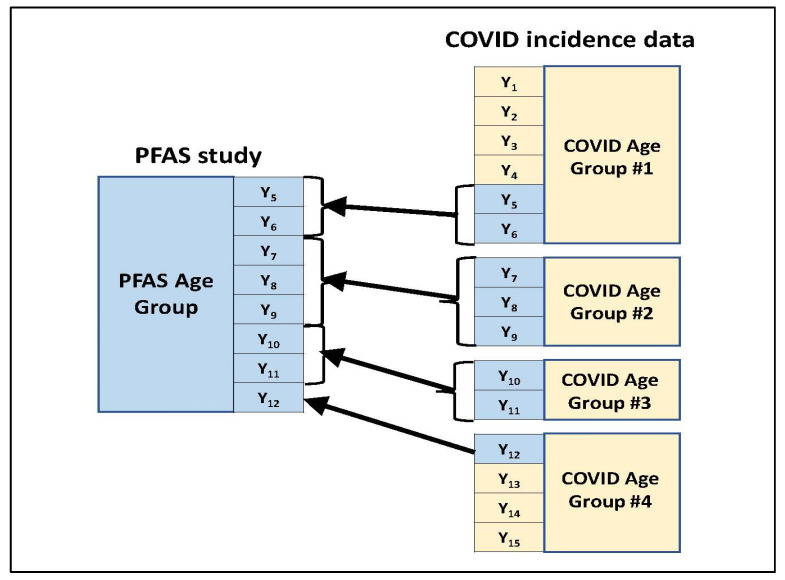
Mapping COVID-19 incidence data age categories to an age category used in the PFAS study.

Ideally, the set of age groups appearing within a study’s blood PFAS concentration summaries (as presented in the study’s publication) would have coincided exactly with the age groups used in reporting COVID-19 incidence rates for the study location on the state’s COVID-19 data dashboard. However, as one may expect, this alignment of age groups did not occur. Therefore, we needed to use the available COVID-19 data to estimate a COVID-19 incidence rate for each of the specific age groups within the publication’s blood PFAS summaries. For a given PFAS study, the approach for estimating these COVID-19 incidence rates involved the following steps:

1. The publicly available COVID-19 incidence count data by age grouping and gender were downloaded for the location in which the participants of the given PFAS study resided. These COVID-19 incidence counts were cumulative since the start of data collection by the State or local agency in charge of acquiring and presenting the COVID-19 data on the dashboard (i.e., total counts were obtained).
  ○ As these data were downloaded, the age groups were delineated for the presented COVID-19 incidence count data.
2. Within each of the COVID-19 incidence age groups encountered in step #1, incidence counts were estimated for each age (year) by dividing the total incidence count for that age group by the number of years represented within the group. This approach assumes that COVID-19 incidence was uniformly spread across the years within that group, and therefore, each year within an age group had the same COVID-19 incidence count estimate.
3. For a given age group present in the blood PFAS concentration summary, those age groups used to present the COVID-19 incidence counts were identified which overlapped with the PFAS age group in some way. Figure 1 illustrates this through an example. In this example, the PFAS study age group spans eight years (Y_5_ to Y_12_) which are represented among four age groups used in the COVID-19 incidence data for the study location. (These four COVID-19 age groups which overlap with the PFAS age group jointly span a total of 15 years—Y_1_ to Y_15_.) Two of the four COVID-19 age groups (#2 and #3) fall within the PFAS age group in their entirety. For each of the other two COVID-19 age groups (#1 and #4), only a subset of their represented years falls within the PFAS age group.
4. Across all years within the given PFAS age group, the year-specific estimated COVID-19 incidence counts calculated in step #2 were summed to obtain an estimated COVID-19 incidence count for that PFAS age group.
5. Steps #3 and #4 were repeated for each age group in the blood PFAS concentration summary, yielding a COVID-19 incidence count estimate for each group.

The estimated COVID-19 incidence counts within each age group present in the blood PFAS concentration summary were divided by the estimated 2019 total population count for that age group within the study’s target population that resides within the study’s location. For example, if a PFAS study targeted a subpopulation consisting of women living in select counties within a given state, then the estimated COVID-19 incidence counts within each age group in the study was divided by the estimated 2019 population for women in that age group who reside in the selected counties. When multiplied by 100%, these results represented the estimated COVID-19 incidence percentages within each age group in the PFAS study.

Upon acquiring the PFAS blood measurement data and COVID-19 incidence data, and implementing the 6-step approach detailed above, the following was obtained for each age group present in the blood PFAS concentration summary within a given PFAS reference:

- Measures for summary statistics related to the distribution of PFAS concentrations in blood (specifically, the 50th and 75th percentiles for both PFOS and PFOA) for the age group.
- COVID-19 incidence measure for the age group (i.e., percentage relative to the group’s population).

This investigation focused on values for the 50th and 75th percentile of PFAS concentrations in blood within a group of study individuals because those percentiles were most frequently reported in the publications for those PFAS studies whose results could be matched with available COVID-19 data from their corresponding states. While other distributional statistics were occasionally reported in the publications (e.g., geometric means, other percentiles), they appeared only sporadically among the publications and may not have been reported by age group, thus not making them amenable for statistical analysis.

Estimated COVID-19 incidence percentages and values for the 50th and 75th percentiles of blood PFAS concentrations were paired for each age group within a given location (stated in the respective PFAS study publication). These paired data were examined across age groups by statistical analysis to assess association between blood PFAS concentration and COVID-19 incidence, and whether age grouping affects this association. As the set of age groupings differed among PFAS studies, the median age within each age category represented the categories in this analysis. When age categories had no lower or upper bound (e.g., 18+ years), the categories in those cases were restated using descriptive terminology, i.e., “young” versus “old” or “youth” versus “adult.”

An analysis of covariance (ANOCOVA) was used to predict the COVID-19 incidence percentage as a function of the blood PFAS concentration percentile value within the population, while accounting for effects of age and the PFAS study on the association. This analysis involved using ordinary least squares techniques to fit a model to the paired data across studies. The blood PFAS concentration percentile value and the median age were treated as continuous predictor variables (associated with a slope factor), while the PFAS study was treated as a categorical predictor variable. As the COVID-19 incidence rate (the variable which the model is predicting) is a percentage from 0–100%, an arcsine square root transformation was applied to these percentages (as a variance stabilizing measure) before fitting the model. If *p* denotes the incidence rate percentage, then this transformation is as follows:

$$Y=\arcsin\left(\sqrt{\frac{p}{100}}\right)$$

Thus, the ANOCOVA model takes the following form:

$$Y_{ij}=\beta_{1}{\left(PFAS\;percentile\right)}_{i}+\beta_{2}{\left(median\;age\right)}_{i}+\gamma_{j}+{\left(error\right)}_{ij}$$

where $\beta_{1}$ is the slope factor applied to the PFAS blood percentile value, $\beta_{2}$ is the slope factor applied to median age, and $\gamma_{j}$ is a constant fixed amount associated with the *j*th PFAS study. If age is categorized (e.g., youth versus adult), then $\beta_{2}\left(median\;age\right)$ is replaced in the model by a fixed age effect similar to the factor denoting the PFAS study. This model was fitted separately for PFOA versus PFOS data and for the specific PFAS percentile considered (50th or 75th).

As seen in the Results section below, the available data across studies were insufficient in number to allow the above model to be fitted to the data (due to overparameterization). Therefore, the above model was reduced by specifying fewer parameters. Specifically, age was included in the model but not study, and vice versa. Each reduced form of the model was then fitted separately to the data. Within each model fitting, Type III F tests were performed at a 0.05 significance level to determine the statistical significance of the model parameters $\beta_{1}$, $\beta_{2}$, and $\gamma_{j}$; if a parameter was found to be statistically significantly different from zero at the 0.05 level, then we concluded that there was a significant association between that variable and COVID-19 incidence rate, after accounting for the other terms in the model. The GLM (general linear model) procedure in SAS^®^ was used to fit the model and perform the F tests.

Of the blood PFAS study references identified for this investigation, the references listed in Table 1 had PFOA and PFOS summary measurements displayed in tables according to age groups. Thus, the publications for these studies included results that were deemed acceptable for use in the statistical analysis, while the results in the publications for other studies contained insufficient data for the analysis. The four studies in Table 1 correspond to studies performed in North Carolina, Ohio, and New Jersey, plus the summary of NHANES data representing the entire nation.

The PFAS measurements presented in the publications noted in Table 1 (50th and 75th percentiles of blood PFAS concentrations among the study cohort) were paired with COVID-19 incidence rates for those same locations, by age groupings. The PFAS incidence data for the study locations in Table 1 were obtained from the following sources:

- New Jersey COVID-19 data: https://covid19.nj.gov/ accessed on 4 February 2022.
- North Carolina COVID-19 data: https://covid19.ncdhhs.gov/dashboard/cases-demographics accessed on 4 February 2022.
- Ohio COVID-19 data: https://coronavirus.ohio.gov/wps/portal/gov/covid-19/dashboards accessed on 4 February 2022.
- NHANES data: https://covid.cdc.gov/covid-data-tracker/#demographics accessed on 4 February 2022.

For Ohio and New Jersey, the COVID-19 data used in the analysis were as posted on 4 September 2021. For North Carolina, the COVID-19 data were as reported through 28 August 2021. Each state’s COVID-19 data are cumulative from the state’s first reported COVID-19 case.

## 4. Results

PFAS blood concentrations within age categories and COVID-19 incidence rates.

Table 2 presents the values for the 50th and 75th percentiles of PFAS blood concentrations within the reported age groupings within these four studies as extracted from their study publications, along with the COVID-19 incidence rates for those states as estimated for the specified age groupings. The Romano study (in southwest Ohio) did not report percentiles by age group for PFOS. For two studies, the age groups could only be classified as “youths” and “adults” when bringing the data together for statistical analysis, while for the other two studies, a median age could be identified for each age group. Thus, because of the age reporting, it was not possible to bring data together across all four of the studies; instead, the analysis was applied to two sets of two studies separately: the Kotlarz study and NHANES, and the Romano and Yu studies.

**Figure 2:**
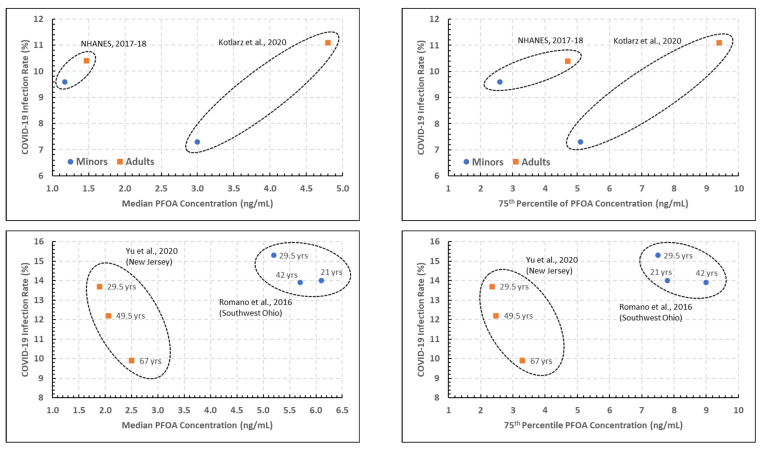
Plots of estimated COVID-19 incidence rate percentage versus the 50th (**left**) and 75th (**right**) percentiles of PFOA blood measurements, denoted by age and PFAS study [5,7,12,14].

The results from the statistical analyses applied to the data in Table 2 are as follows:

**PFOA.**Figure 2 presents scatterplots of the estimated COVID-19 incidence rates versus the 50th (median) and 75th percentiles of the measured blood PFOA concentrations, all as presented in Table 2, with the age groupings and studies noted within the plots.

The two top plots of Figure 2, corresponding to the 2017–2018 NHANES and the North Carolina study, show a considerable difference in PFOA blood concentration percentile between the two studies, and noticeable increases in both the PFOA blood concentration and COVID-19 incidence rates for adults versus youths. The statistical analysis performed on the data in the top two plots of Figure 2 indicated that the association between COVID-19 incidence rates and both the PFOA concentration percentiles and study were significant at the 0.05 level (*p* = 0.021 to 0.025), while the association with age grouping (youths versus adults) was not significant in either case.

The two bottom plots of Figure 2, presenting data from the Ohio and New Jersey studies, tell a somewhat different story. While the two studies are separated in their PFOA blood concentration percentiles as in the studies in the top plots, there are not clear trends with age groups in either study with regard to either PFOA concentration percentile or COVID-19 incidence rates. In addition, while there are slightly more data points in this analysis (6, versus 4 in the analysis for the top two plots), the statistical analysis did not result in a statistically significant association with the COVID-19 incidence rates (*p* = 0.055 to 0.124 for the association with age; *p* > 0.10 for the association with the PFOA concentration percentile). In part, this is due to the preliminary nature of this analysis with very limited data.

**Figure 3:**
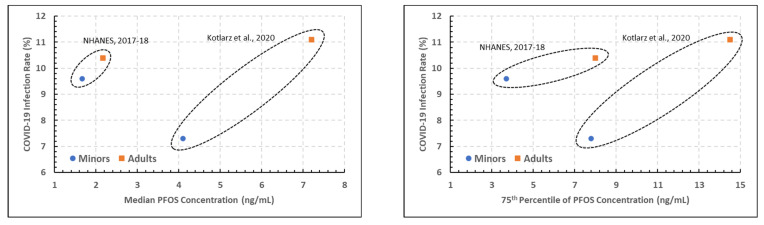
Plots of estimated COVID-19 incidence rate percentage versus the 50th (**left**) and 75th (**right**) percentiles of PFOS blood measurements, denoted by age and PFAS study [5,12].

**PFOS.**Figure 3 presents two scatterplots of the estimated COVID-19 incidence rates versus the 50th (left) and 75th (right) percentiles of the measured blood PFOS concentrations presented in Table 2 for the North Carolina and NHANES studies (i.e., the PFOS versions of the top two PFOA plots in Figure 2). The patterns are very similar to those seen for PFOA for these two studies. However, the statistical analysis did not result in a significant association between COVID-19 and either PFOA concentration or study at a 0.05 level (*p* > 0.10). This lack of statistical significance could be the result of a small number of data points entering into the analysis.

At the 50th percentile (median), age groups having PFOS concentrations appear to have had less COVID-19 in the 20–39 age group versus the 25–34 age group. Given the cut-off ages for each group, it is likely that a younger age group inclusion helped to show less COVID-19 cases.

At the 75th percentile of PFOS concentration within age groupings, COVID-19 cases are seen to increase with each increasing age group from the respective population.

## 5. Discussion

Comparison between NHANES and North Carolina studies (Figure 2) revealed a considerable difference in PFOA blood concentration percentile, with noticeable increases in PFOA blood concentration and COVID-19 incidence rates for adults versus youths. Association between COVID-19 incidence rates and both the PFOA concentration percentiles and study were significant, while age grouping (youths versus adults) was not significant. Comparison between Ohio and New Jersey studies did not demonstrate significance with age for either PFOA concentration percentile or COVID-19 incidence rates. Estimated COVID-19 incidence rates versus the 50th and 75th percentiles of blood PFOS concentrations presented in the North Carolina and NHANES studies (Figure 3) were similar to those for PFOA (above). At the 50th percentile, age groups having PFOS concentrations appear to have had less COVID-19 in the 20–39 age group versus the 25–34 age group. At the 75th percentile of PFOS concentration within age groupings, COVID-19 cases are seen to increase with each increasing age group from the respective population.

A general trend observed is for PFAS (PFOA and PFOS) levels to be higher in older age groups [14,17]. This was supported by our observation in the 75th percentile groupings for PFOA and PFAS. Further, a younger age group should demonstrate fewer COVID-19 cases, as was also determined. While this does not determine causality, such associations should help to promote further study.

Interestingly, significant associations were observed between PFAS serum concentrations and age (to a 20+ year old age group) in a New Hampshire drinking water study with a comparison to 2013-2014 NHANES data [18]. Increased PFAS levels associated with age have also been found in a New York state study of older adults, where 25% higher serum PFOS and 80% higher PFOA levels in study participants compared to NHANES data [19]. Increasing age was also found to be associated with higher PFAS levels in a study of male anglers in Wisconsin [17].

While PFOA and PFOS are no longer being made in the U.S., they continue to be manufactured in other nations, and continued exposures are possible from these sources and from other PFAS [20,21]. Additionally, an overall decrease in blood PFAS levels in the national population in recent years is anticipated, given the decrease in PFAS use and dissemination [22]. According to the ATSDR: “Since 2002, production and use of PFOS and PFOA in the United States have declined. As the use of some PFAS has declined, some blood PFAS levels have gone down as well. From 1999 to 2014, blood PFOS levels have declined by more than 80% and blood PFOA levels have declined by more than 60%.” [22].

Concurrently, COVID-19 mortality in the US and in much of the world has been highest in elderly populations. For example, in 50–64 year olds, the risk for hospitalization from COVID-19 has been 4 times that of 18–29 year olds; for 65–74 year olds, the risk is 6 times. Similarly, for 50–64 year olds, the risk for death from COVID-19 has been 35 times that of 18–29 years olds, and for 65–74 year olds, that risk has been an astounding 95-fold increased mortality [23]. Multiple comorbidities associated with increasing age greatly contribute to severity of disease and adverse clinical outcomes [24]. A recent Italian study found a higher COVID-19 mortality risk among a population heavily exposed to PFAS [25]. Potential PFAS exposure through drinking water and its resulting effects on the immune system are significant concerns for achieving COVID-19 illness [26].

While it is important not to infer causality from the higher levels of PFAS observed in elderly populations that also have higher serious adverse events from COVID-19 infections, it is also reasonable to assume that these events are not wholly unrelated. PFAS exposures occurring early in life may cause deleterious health effects later in life. According to the ATSDR: “CDC/ATSDR recognizes that exposure to high levels of PFAS may Plesimpact the immune system. There is evidence from human and animal studies that PFAS exposure may reduce antibody responses to vaccines [27,28], and may reduce infectious disease resistance [29]. Because COVID-19 is a new public health concern, there is much we don’t know. More research is needed to understand how PFAS exposure may affect illness from COVID-19.”

## 6. Conclusions

Elderly populations in the U.S. bear the greatest burden of hospitalization and death from COVID-19 infection. It is less clear what percent of that morbidity and mortality is attributable to prior lifetime PFAS exposure. However, due to historic exposure to PFAS, elderly populations in certain geographic locations may bear the brunt of lifetime exposure. Further, discrete, continuing, or intermittent PFAS exposure over a lifetime could be one contributing factor to increased COVID-19 vulnerability among an elderly population.

Ongoing efforts to continue to reduce PFAS exposures nationally may not directly affect a group that previously had significant lifetime PFAS exposure. It is therefore important to better understand the nature of the relationship between PFAS exposure and COVID-19 infection through ongoing biomonitoring and environmental assessment studies. Our examination of the data from recent studies shows that the highest levels of both cumulative PFAS exposure and severe health outcomes from COVID infection are found in the oldest populations for which such data exists.

## Figures and Tables

**Table 1:** List of references providing PFOA and/or PFOS blood concentration data summaries by age group and deemed acceptable for the statistical analysis.

Reference #	Study Name/Location	Target Population	Sample Collection Period	# Study Participants
[12]	GenX Exposure Study	Residents ≥6 years of age in New Hanover County, NC, USA	2017–2018	344
[7]	Health Outcomes and Measures of the Environment (HOME) Study (pregnant women recruited)	Females of child-bearing age residing in Butler, Clermont, Hamilton, and Warren counties in Southwest Ohio	2003–2005	336
[14]	New Jersey Biomonitoring (NJBM) Study	New Jersey residents from 20 to 74 years of age	2016–2018	1030
[5]	NHANES	U.S. population	2017–2018	1929

**Table 2:** 50th and 75th percentiles of PFOA and PFOS blood concentration measures, and estimated COVID-19 incidence rates for the given age group, for each study included in statistical analysis.

Study (Reference #)		Age Group	Age Category or Median Age (years)	COVID-19 Incidence Rate	PFOA Concentration Percentiles (ng/mL)		PFOS Concentration Percentiles (ng/mL)	
					50th	75th	50th	75th
[12]	NC	06–17 years.	Youths	7.3%	3.00	5.10	4.10	7.8
[12]	NC	18+ years.	Adults	11.1%	4.80	9.40	7.20	14.5
[5]	Nation	12–19 years.	Youths	9.6%	1.17	2.60	1.67	3.7
[5]	Nation	20+ years.	Adults	10.4%	1.47	4.70	2.17	8.0
[7]	OH	18–24 years	21 years	14.0%	6.10	7.80	--	--
[7]	OH	25–34 years	29.5 years	15.3%	5.20	7.50	--	--
[7]	OH	35–49 years.	42 years	13.9%	5.70	9.00	--	--
[14]	NJ	20–39 years.	29.5 years	13.7%	1.89	2.35	2.67	3.70
[14]	NJ	40–59 years.	49.5 years	12.2%	2.06	2.48	2.98	4.19
[14]	NJ	60–74 years.	67 years	9.9%	2.50	3.29	4.46	6.12

## Data Availability

Data used for analysis is publicly available.
